# Study on the Synergetic Fire-Retardant Effect of Nano-Sb_2_O_3_ in PBT Matrix

**DOI:** 10.3390/ma11071060

**Published:** 2018-06-22

**Authors:** Lei Niu, Jianlin Xu, Wenlong Yang, Jiqiang Ma, Jinqiang Zhao, Chenghu Kang, Jiaqiang Su

**Affiliations:** 1 State Key Laboratory of Advanced Processing and Recycling of Non-ferrous Metals, Lanzhou University of Technology, Lanzhou 730050, China; niulei@lut.cn (L.N.); ywl2414@126.com (W.Y.); majq@lut.cn (J.M.); lutzhaojin@163.com (J.Z.); kchlut111@163.com (C.K.); 2 Baiyin Research Institute of Novel Materials, Lanzhou University of Technology, Baiyin 730900, China; 3 Lanzhou Industry Research Institute, Lanzhou 730000, China; sujiaqiang10@163.com

**Keywords:** Nano-Sb_2_O_3_, poly(butylene terephthalate), thermostability, flammability

## Abstract

Nano-Sb_2_O_3_ has excellent synergistic flame-retardant effects. It can effectively improve the comprehensive physical and mechanical properties of composites, reduce the use of flame retardants, save resources, and protect the environment. In this work, nanocomposites specimens were prepared by the melt-blending method. The thermal stability, mechanical properties, and flame retardancy of a nano-Sb_2_O_3_–brominated epoxy resin (BEO)–poly(butylene terephthalate) (PBT) composite were analyzed, using TGA and differential scanning calorimetry (DSC), coupled with EDX analysis, tensile testing, cone calorimeter tests, as well as scanning electron microscopy (SEM) and flammability tests (limiting oxygen index (LOI), UL94). SEM observations showed that the nano-Sb_2_O_3_ particles were homogeneously distributed within the PBT matrix, and the thermal stability of PBT was improved. Moreover, the degree of crystallinity and the tensile strength were improved, as a result of the superior dispersion and interfacial interactions between nano-Sb_2_O_3_ and PBT. At the same time, the limiting oxygen index and flame-retardant grade were increased as the nano-Sb_2_O_3_ content increased. The results from the cone calorimeter test showed that the peak heat release rate (PHRR), total heat release rate (THR), peak carbon dioxide production (PCO_2_P), and peak carbon monoxide production (PCOP) of the nanocomposites were obviously reduced, compared to those of the neat PBT matrix. Meanwhile, the SEM–energy dispersive spectrometry (EDX) analysis of the residues indicated that a higher amount of C element was left, thus the charring layer of the nanocomposites was compact. This showed that nano-Sb_2_O_3_ could promote the degradation and charring of the PBT matrix, improving thermal stability and flame retardation.

## 1. Introduction

With the development of lightweight-oriented automobiles, poly(butylene terephthalate) (PBT) engineering plastics has become a focus of attention in the development and application of automobiles [1]. However, neat PBT can easily burn if ignited, with a peak of heat release rate up to 1404 kw/m^2^. A large amount of heat would be released in the combustion process, which would cause increased fire accident fatalness [2]. Therefore, the development of PBT composite with comprehensive mechanical properties, flame retardancy, and cost-effectiveness has become a key to expand PBT application.

Halogen flame retardants are widely used because of their high flame retardancy efficiency and low price. However, halogen flame retardants release toxic, corrosive gas and pollute the environment, which restrict their application. Carbon nanotubes and graphene has become a research focus [3,4], but their complex preparation and high cost made them difficult to industrialize. Large additive amount of phosphorus compounds, metal hydroxides, and organic montmorillonite deteriorate the general physical and mechanical properties of matrix materials and restrict their further application. To meet the needs of practical applications of polymer composites, halogen-free flame retardants, nano-flame retardants and traditional halogen flame retardants were used together [5] in order to solve the key problem in practical application of decreasing the amount of halogen flame retardants while maintaining the flame retardancy and the comprehensive physical and mechanical properties of the polymer composites.

The present research on flame retardancy of PBT matrix material is mainly focused on additive flame retardants, which include flame retardants containing phosphorus, halogen–antimony synergetic flame retardants, and different flame retardants used together. The dispersion performance of the flame retardants [6], structure and properties of carbonized production [7], melting and dropping production [8], degradation kinetics [9], and correlation of flame retardancy for the PBT composites and retardant mechanism were investigated. For example, phosphorous flame retardants synergistic montmorillonite [10], Sb_2_O_3_ [11], and carbon nanotubes [12] flame retardant PBT matrix materials produced catalytic crosslinking in condensed phase, by which thermal stability and residual carbon content increased. Meanwhile, the combustible volatile products were diluted, the free radicals in the gas phase were captured, and the gas phase and the condensed phase worked together to produce the effect of flame retardancy. A study found that nano-Sb_2_O_3_ particles exhibit excellent comprehensive mechanical properties and flame retardancy [13] in the same condition. In view of this, the preparation of nano-Sb_2_O_3_ particles [14,15], their surface modification [16], and synergistic flame retardancy were researched, promoting the application and development of nano-Sb_2_O_3_. When nano-Sb_2_O_3_ and halogen flame retardants [17], metal hydroxide [18], carbon nanotubes [19] were used together to strengthen the flame-retardant efficiency of composites, the heat release rate and the total heat release rate were obviously reduced, and the residue increased significantly.

In summary, characterization methods such as differential scanning calorimetry (DSC), TGA, SEM, and cone calorimeter were used to investigate the crystallization behavior, thermal stability, mechanical properties, and flame retardancy. The dispersion performance of the nano-Sb_2_O_3_ particles in PBT composites and the residual carbon morphology after combustion were analyzed by SEM-equipped energy dispersive spectrometry (EDX). On this foundation, synergistic flame retardancy of nano-Sb_2_O_3_ and brominated epoxy resin (BEO) were further studied.

## 2. Experimental

### 2.1. Materials

Poly(butylene terephthalate) (PBT, 1100-211M), with a density of 1.31 g/cm^3^, was provided by Taiwan Chang Chun Plastics Co., Ltd., Suzhou, China. Nano-Sb_2_O_3_ were prepared and modified by ball milling, as reported in our study [20]. The particle size was 50–100 nm. Brominated epoxy resin (BEO, with an average weight of 20,000 and a bromine content of 53.2%) was provided by BASF Chemical Co., Shanghai, China.

### 2.2. Preparation of Nanocomposites

First, PBT powder, BEO powder, and nano-Sb_2_O_3_ particles mixtures were dispersed by high-energy ball milling. The ball grinding speed was 400 r/min, and the ball grinding time was 6 h. The mixtures were dried at 110 °C overnight in a vacuum oven before use. Second, nanocomposites were prepared with the dried mixtures through melt blending in a twin-screw extruder (SJZS-10A, Wuhan Ruiming Plastic Machinery Co., Wuhan, China), and the barrel temperatures were set at 225 °C, 230 °C, 240 °C, and 245 °C, respectively. Finally, samples were obtained by microinjection molding (SZS-20, Wuhan Ruiming Plastic Machinery Co., Wuhan, China). The sample material ratio is shown in Table 1. The mass ratios of the specimens (Br/Sb) were 9.8, 3.3, and 2.0.

### 2.3. Characterization

At room temperature, tensile tests of the PBT matrix and its nanocomposites were conducted with a WDW-500E tensile test machine (Nanjing Time Instrument Co., Ltd., Nanjing, China) at a speed of 20 mm/min. The average of the five individual determinations was calculated.

The surface morphology of fracture and carbonization were characterized by means of scanning electron microscopy (SEM). Energy dispersive spectrometry (EDX) analyses were performed to study the dispersion of nano-Sb_2_O_3_ particles in the PBT matrix at a high magnification. All samples were coated with a thin homogenous gold layer by ion-sputtering to facilitate the measurements.

Differential scanning calorimetry (DSC) was used to analyze the samples’ thermal properties. Scanning was performed from room temperature to 300 °C with a heating rate and a cooling rate of 10 °C/min under a nitrogen atmosphere.

A thermogravimetric analysis (TGA) instrument was used to measure the thermal decomposition behaviors under nitrogen. The sample mass was in the range of 5–10 mg. The samples in an open alumina crucible were heated from room temperature to 600 °C at a linear heating rate of 10 °C/min.

The limiting oxygen index (LOI) values were determined by a limiting oxygen index instrument, according to GB/T 2406.2-2009 [21] (sample dimensions 80 × 10 × 4 mm^3^). A UL-94 vertical burning test was carried out with a CZF-3 instrument, according to GB/T 2408-2008 (sample dimensions 125 × 12.5 × 3.2 mm^3^).

The fire behavior under forced-flaming conditions was assessed using a cone calorimeter. The tests were performed according to the ISO 5660 standard [22]. The specimens (100 × 100 × 15 mm^3^) were measured in aluminum foil and exposed horizontally to an external heat flux of 50 kW/m^2^ from the heating coils, in well-ventilated conditions (air rate 24 L/s). The residues were collected after the test and subsequently analyzed by SEM coupled with EDX.

## 3. Result and Discussion

### 3.1. Crystallization and Melting Behavior

**Figure 1:**
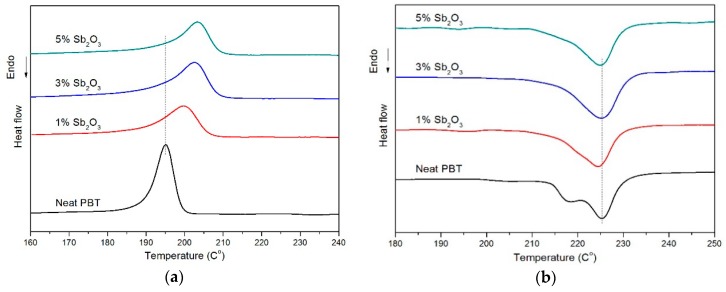
DSC non-isothermal crystallization curves (**a**) and melting curves (**b**) of neat poly(butylene terephthalate) (PBT) and its nanocomposites.

The non-isothermal crystallization and melting behaviors of neat PBT and its nanocomposites are presented in Figure 1. The obtained thermodynamic parameters of crystallization and melting behavior, such as the initial crystallization temperature (T_onset_), the crystallization peak temperature (T_c_), the crystallization rate of the polymer (T_onset_-T_c_), the enthalpy of melting (ΔH_m_), the nucleation efficiency of the polymer (NE),a and the crystallinity (X_c_) are listed in Table 2.

The degree of crystallinity of neat PBT was calculated from the melting enthalpies by the following Equation (1) [23]:

(1) $$X_{c}=\frac{\Delta H_{m}}{\Delta H_{m}^{0}}\times 100\%$$

where $\Delta H_{m}$ is the melting enthalpy of the samples, and $\Delta H_{m}^{0}$ is the heat of fusion corresponding to 100% crystalline PBT, which is 140 J/g [24]. The degree of crystallinity for nanocomposites was then determined by considering the weight fraction of the PBT matrix (W_f_), by the following Equation (2).

(2) $$X_{c}=\frac{{\Delta H}_{m}}{{\Delta H}_{m}^{0}\times W_{f}}\times 100\%$$

The nucleation efficiency of the nanocomposites was calculated by the following Equation (3)

(3) $$\mathrm{NE}=\frac{\left(T_{\mathrm{ca}}{-T}_{c1}\right)}{\left(T_{c2}{-T}_{c1}\right)}\times 100\%$$

where T_ca_ is the crystallization temperature after adding the nucleating agent, T_c1_ is the crystallization temperature of neat PBT, and T_c2_ is the highest crystallization temperature of the system self-nucleation.

It is shown in Table 2 that the addition of nano-Sb_2_O_3_ particles increased the crystallinity temperature and nucleation efficiency of the nanocomposites and showed regularity. With the increase of the mass fraction of the nano-Sb_2_O_3_ particles, the crystallization temperature and nucleation efficiency increased, but the rate of this increase diminished. This was due to the effect of nano-Sb_2_O_3_ on PBT with heterogeneous nucleation. The increase of the crystallization temperature slowed down the nucleation efficiency, which indicated that heterogeneous crystal nuclei increased with the increase of the nano-Sb_2_O_3_ mass fraction, and nano-Sb_2_O_3_ particles dispersed difficultly and began to agglomerate. The number of heterogeneous crystal nuclei reached the limit; with the increase of the nano-Sb_2_O_3_ mass fraction, it first decreased and then rose. T_onset_-T_c_ gradually decreased and then rose with the increase of the mass fraction of nano-Sb_2_O_3_, which indicated that the crystallization rate and crystallinity of PBT raised and then decreased. This was the result of interfacial interactions between nanoparticles and PBT, strengthened after nano-Sb_2_O_3_ was added by surface treatment. The free energy of the nucleation process was reduced, thus the PBT segment was easy to adsorb and nucleate. When PBT started to crystallize at a higher temperature, more perfect and stable crystals were formed, which was beneficial to the improvement of mechanical strength as well as to the reduction of multiple melting behavior. Nucleation was also beneficial to regular stacking and crystallization of PBT molecular chains [25]. The degree of crystallinity increased, and the grain was refined. However, when the addition of nanoparticles reached a certain value, the particles as centers of the induction of crystallization gradually became saturated. The function of the nanoparticles as nucleating agents gradually reduced and the crystallinity of the composite system began to reduce. This is consistent with the crystallization rule of carbon-based nanofillers/PBT composites studied by Huajie Yin et al. [26].

The curves of DSC non-isothermal crystallization are shown in Figure 1a. The curve of *T_c_* for nano-Sb_2_O_3_–PBT composites showed a remarkable increase of more than 8 °C at the highest nano-Sb_2_O_3_ loading level (i.e., 5 wt %). This result showed that the introduction of nano-Sb_2_O_3_ accelerated the crystallization process of PBT through a heterogeneous nucleation effect. In the following heating scan, multiple melting behaviors were observed in the neat PBT, as shown in Figure 1b. The multiple melting behaviors were caused by fusion of a certain amount of the original crystals, followed by recrystallization, and final melting of more perfect crystals, which partly formed during the primary crystallization and partly formed through the recrystallization process occurred during the heating scan [27]. Conversely, the nano-Sb_2_O_3_/PBT composites showed single-melting behavior. Nano-Sb_2_O_3_ acted as an additional active substrate, which promoted the crystallization of the PBT matrix. This heterogeneous nucleation also led to the formation of more defect-ridden crystalline lamellae and less ordered PBT crystals [28], which possessed a single-melting behavior as a result. The presence of the nano-Sb_2_O_3_ reduced the ability of the polymer chains to be fully incorporated into the growing crystalline lamellae to some extent.

### 3.2. Mechanical Properties

The mechanical properties of the PBT matrix and its nanocomposites are shown in Table 3. It can be seen that the tensile strength and Young’s modulus of the nanocomposites became better than those of the PBT matrix when adding to nano-Sb_2_O_3_ particles. The tensile strength was increased by increasing the nano-Sb_2_O_3_ particles content from 1 wt % to 3 wt %, because the modifying agent enhanced the interfacial adhesion between the nano-Sb_2_O_3_ particles and the PBT matrix, which made the nano-Sb_2_O_3_ particles absorb certain loads in the tension process. On the other hand, the improvement of grain refinement and crystallinity of the PBT removed stress concentration, which was caused by ununiform grain size distribution during the tensile process. The ability to resist deformation clearly improved. This showed that the tensile strength of the composites was increased, and then the mechanical properties of PBT could be improved; however, they began to decrease when the nano-Sb_2_O_3_ particles content reached 5 wt %. The decrease of the tensile strength was due to the poor dispersion of the nano-Sb_2_O_3_ particles in the PBT matrix and the low level of interfacial adhesion between the two components. The Young’s modulus of the nanocomposites increased with the nano-Sb_2_O_3_ particles content and then slightly decreased at 5 wt %.

**Figure 2:**
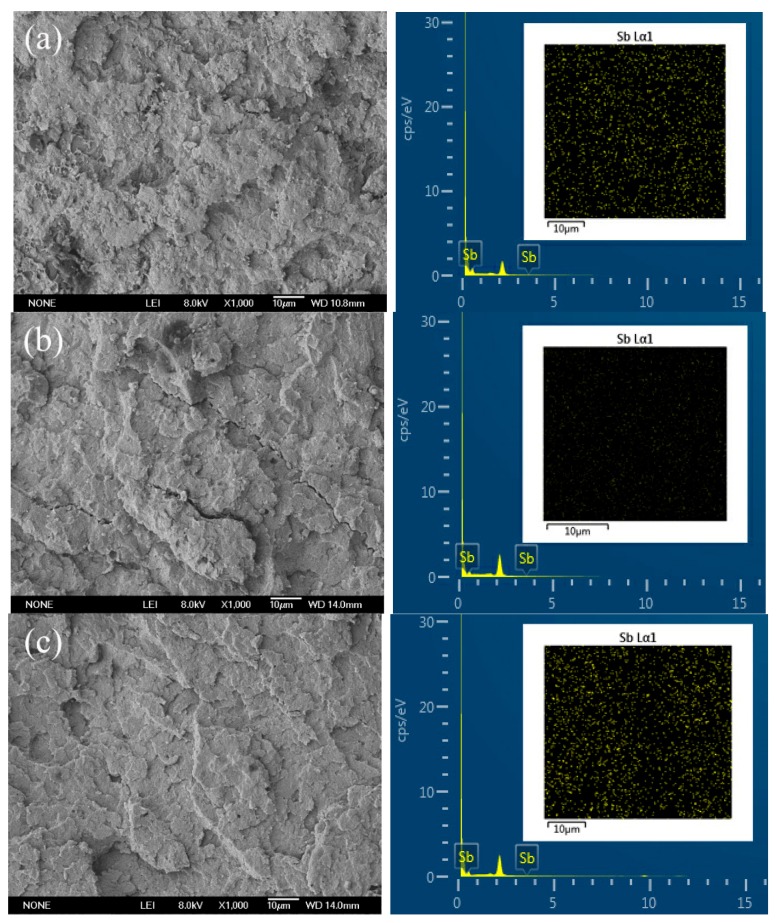
Scanning electron microscopy (SEM) micrographs and energy dispersive spectrometry (EDX) images of nanocomposites fractured surfaces. (**a**) 1 wt % nano-Sb_2_O_3_ composites, (**b**) 3 wt % nano-Sb_2_O_3_ composites, and (**c**) 5 wt % nano-Sb_2_O_3_ composites.

The SEM micrographs and EDX images are shown in Figure 2. The dispersion of the nano-Sb_2_O_3_ particles in the PBT matrix was revealed by EDX. It was observed that nano-Sb_2_O_3_ particles were dispersed uniformly in the PBT matrix. However, the agglomeration tendency of the nano-Sb_2_O_3_ particles in the PBT matrix gradually augmented with the increase of the nano-Sb_2_O_3_ particles content. As a result, the nanocomposites presented low tensile strength at the highest nano-Sb_2_O_3_ particles content. The nanocomposites containing 5 wt % nano-Sb_2_O_3_ particles had relatively smooth appearances (Figure 2c). When the nano-Sb_2_O_3_ particles content was 1 wt % and 3 wt %, the tensile fractured surfaces showed a rough region and cracks (Figure 2a,b). Less nano-Sb_2_O_3_ particles could act as lower stress concentrators [29] in the nanocomposites, which could absorb more energy to improve the tensile strength, consistently with the tensile test results.

### 3.3. Thermal Decomposition Behaviors

**Figure 3:**
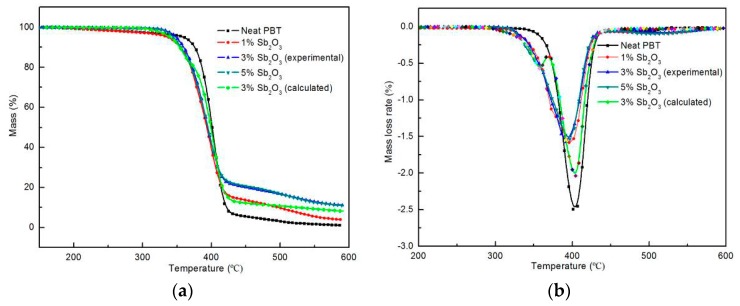
TGA (**a**) and DTG (**b**) curves of the neat PBT matrix and its composites under nitrogen.

The thermal degradation behaviors of the neat PBT and its composites were investigated by TGA in N_2_. The mass loss and mass loss rate curves of the neat PBT and its composites are displayed in Figure 3, and the related data are summarized in Table 4. T_10%_ was defined as the temperature at which the weight loss was 10 wt %. T_Peak%_ was the maximum value of the weight loss. As we could see, the decomposition of the neat PBT and its composites could be divided into two stages: a fast decomposition stage and a stable carbon layer slow decomposition stage. The first stage occurred mainly between 330 and 430 °C. In this stage, the decomposition temperature range of the neat PBT matrix was narrow, concentrated at 370–430 °C, with a 10% weight loss that occurred at 372 °C, a maximum mass loss rate at 407 °C, and a weight loss as high as 91%. The initial decomposition temperature and peak heat release rate of PBT decreased after adding the nano-Sb_2_O_3_ flame retardant. This effect might be attributed to the catalytic effect of the metal oxide on the fragmentation of the macromolecule chain [30], which promoted the rapid oxidation of PBT. This demonstrated that an interaction between the flame retardant and PBT promoted the flame-retardant composites to degrade at lower temperatures, resulting in some high-quality residual char layer.

In the second stage, as the temperature continued to rise, the initial carbon layer decomposed gradually, but the residues of composite were higher compared with the neat PBT. It has been reported that an efficient charring process in flame-retardant polymeric materials occurred at a temperature higher than the processing temperature but much lower than the decomposition temperature of the polymer matrix [31]. As a result, this earlier weight loss in the first degradation step was favorable to retard the degradation of the polymeric matrix in a higher temperature range. The neat PBT left only 1.1 wt % char residue at 600 °C, while the addition of nano-Sb_2_O_3_ resulted in the improvement of the char yields of PBT. When the nano-Sb_2_O_3_ content was 1 wt %, 3 wt %, and 5 wt %, the char yields content at 600 °C was 2.9 wt %, 11.8 wt %, and 10.1 wt %, respectively. Moreover, the experimental value of char residues for the 3 wt % nano-Sb_2_O_3_ composites at 600 °C was higher than the calculated value. These results showed that the components of the nanocomposites interacted with each other, and the presence of nano-Sb_2_O_3_ could significantly enhance the thermal stability of the nanocomposites.

### 3.4. Fire Behaviour: Forced Flaming Combustion (Conecalorimeter)

The flame retardation properties of the neat PBT and its composites were investigated by combustion calorimetry under a forced combustion to obtain thermal combustion data, including heat release rate (HRR), peak heat release rate (PHRR), total heat release (THR), CO production (COP), CO_2_ production (CO_2_P), smoke production rate (SPR), total smoke production (TSP), and so on. The related data are summarized in Table 5.

**Figure 4:**
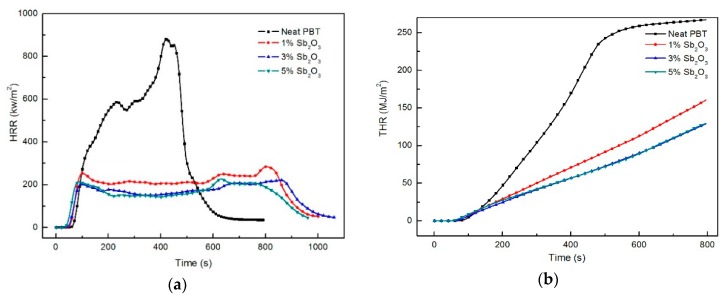

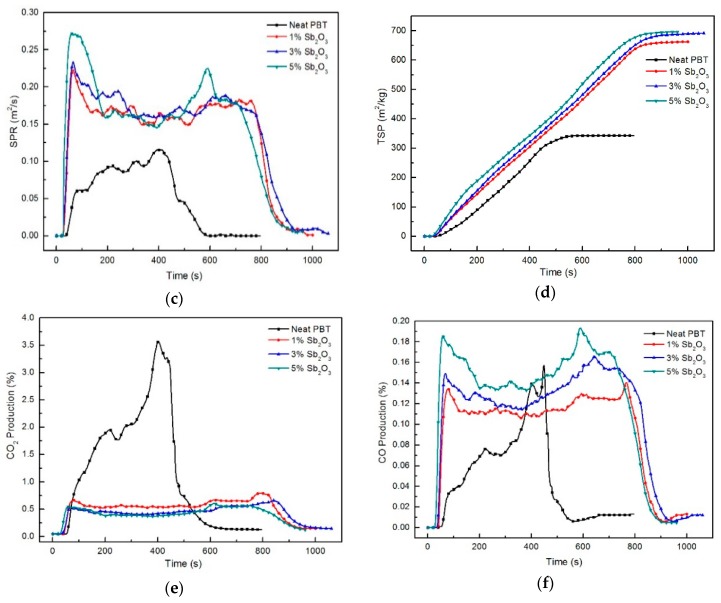
(**a**) Heat release rate (HRR); (**b**) total heat release rate (THR); (**c**) smoke production rate (SPR); (**d**) total smoke production (TSP); (**e**) CO_2_ production; (**f**) CO production as a function of the burning time for the neat PBT and its composites in the cone calorimeter tests at 50 kW/m^2^.

**Figure 5:**
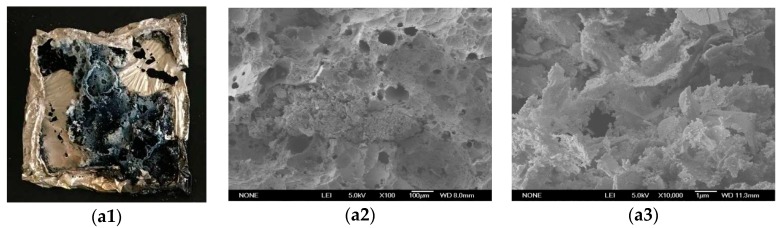

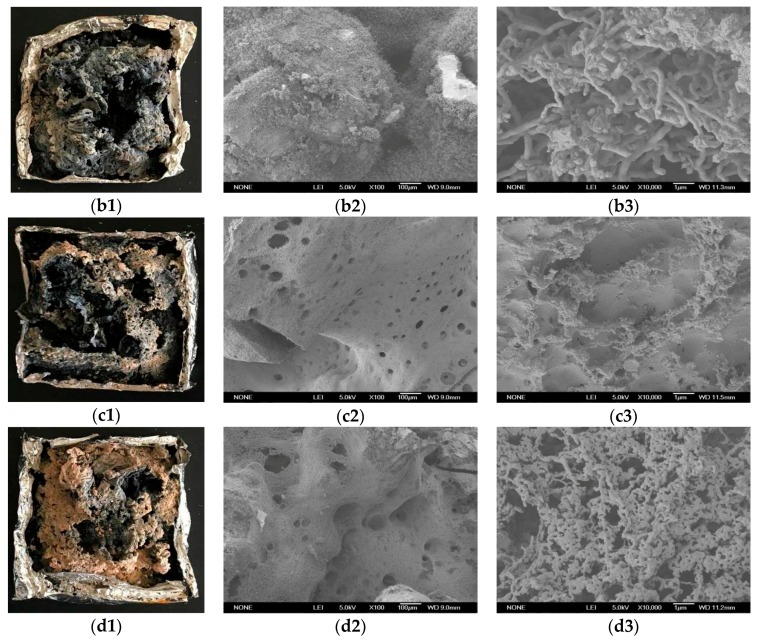
Digital photographs and SEM images of cone calorimeter residues of the neat PBT and its composites: (**a**) neat PBT; (**b**) 1 wt % nano-Sb_2_O_3_ composites; (**c**) 3 wt % nano-Sb_2_O_3_ composites; (**d**) 5 wt % nano-Sb_2_O_3_ composites, in the cone calorimeter tests at a heat flux of 50 kW/m^2^.

HRR, PHRR, and THR are very important parameters to evaluate the combustion performance of polymers, which could be used to predict the transferring speed of flame and fire size. As shown in Figure 4a,b and Table 5, the HRR rose rapidly when the neat PBT had been burnt and decreased rapidly after the vertical displacement reached the peak (917.5 kW/m^2^). By increasing the amount of the nano-Sb_2_O_3_ flame retardant, the value of HRR and THR showed an obvious decrease for the nanocomposites. When the content of the nano-Sb_2_O_3_ flame retardant exceeded 3 wt %, the HRR value did not change much. The HRR, PHRR, and THR of the neat PBT were 375.1 kW/m^2^, 917.5 kW/m^2^, and 265.6 kW/m^2^, respectively. The amount of flame retardants added to nano-Sb_2_O_3_ was 1 wt %, 3 wt %, and 5 wt %. In these conditions, HRR, PHRR, and THR were significantly reduced, with maximum decreases of 57.6%, 75.5%, and 43.7%. This showed that nano-Sb_2_O_3_ could reduce the release of heat during the combustion process of the composite materials. As shown in Figure 5, nano-Sb_2_O_3_ promoted the formation of a protective carbon layer on the surface of the composite in the combustion process. The formation of a carbon layer prevented heat transfer and flame transmission to a certain extent [32], retarded the combustion of the bottom polymer, inhibited the growth of HRR and THR, and extended the combustion time. In addition, as can be seen from Figure 4b, the curve inclination rate of THR decreased, which indicates that that fire spread rate could be reduced by the addition of nano-Sb_2_O_3_.

SPR and TSP are also important parameters when evaluating flame retardancy [33]. As shown in Figure 4c,d and Table 5, the SPR and TSP peak values were significantly higher after adding nano-Sb_2_O_3_. The SPR and TSP peak values of the neat PBT were 0.073 m^2^/s and 342 m^2^/kg, respectively. Adding different nano-Sb_2_O_3_ contents of 1 wt %, 3 wt %, and 5 wt %, the peak values of SPR were 0.21 m^2^/s, 0.241 m^2^/s, and 0.33 m^2^/s, and the peak values of TSP were 662 m^2^/kg, 691 m^2^/kg, and 698 m^2^/kg. This indicated that the addition of nano-Sb_2_O_3_ could inhibit the release of heat and increase the flue gas release. This was due to gas phase flame retardancy and solid phase flame retardancy produced by the bromine–antimony synergistic flame retardant, further preventing air from entering the surface of the specimens. Heat and mass transfer between polymer and heat source were limited, which resulted in incomplete combustion of the bottom polymer. This was also the main reason for the increase of smoke production.

Carbon dioxide (CO_2_) and carbon monoxide (CO) toxic gases produced in a fire are a chief cause of asphyxiation. As shown in Figure 4e,f and Table 5, the release curves of CO_2_ and carbon monoxide of the neat PBT are similar to the HRR and SPR curves, respectively. After adding nano-Sb_2_O_3_ flame retardants, CO_2_ emission reduced observably. By adding different nano-Sb_2_O_3_ contents of 1 wt %, 3 wt %, and 5 wt %, CO_2_ emission were reduced by 65.7%, 68%, and 76.7%, and CO emission were reduced by 35.4%, 8.8%, and 10.1%, respectively. This showed that the addition of nano-Sb_2_O_3_ flame retardants had the role of a flame inhibitor in the gas phase. The nano-Sb_2_O_3_ flame retardants could effectively reduce the production of CO and CO_2_ in the combustion process, which has a great significance in fire avoidance.

In order to investigate the flame retardant mechanism, the structures of these residues were characterized by SEM coupled with EDX. The digital photographs and SEM images of the residues for the neat PBT and its composites are displayed in Figure 5. Here, Figure 5(a1–a3) were macroscopic feature, low and high magnification of SEM images after burning, respectively. Representing method in Figure 5b,c were same as 5a. As it could be seen from Figure 5, there appeared to be more solid chars left behind on the surface of the nanocomposites than on that of the neat PBT. This could be explained by the better carbonization effects of nano-Sb_2_O_3_ on the PBT matrix, in agreement with the TGA analysis and cone calorimeter tests. The EDX results of the residues for the neat PBT and its composites were summarized in Table 6. The residue samples of the neat PBT and the 1 wt % nano-Sb_2_O_3_ composites were primarily composed of C and O elements. The residues were only composed of C element when the content of nano-Sb_2_O_3_ were 3 wt % and 5 wt %. All composites were free of Sb element. This showed that Sb element decomposed from the composites participated in the gas-phase flame retardant action. The Br–Sb synergistic flame-retardancy system could effectively trap radicals in the carbonization reaction. Figure 5c,d showed that the composites residue was firmer, thicker, and more compact. The more compact carbon layer could effectively insulate the air and act as an effective barrier to prevent heat transfer, protecting the underlying materials from further burning [34], which effectively reduces the fire hazards.

### 3.5. Flammability

**Figure 6:**
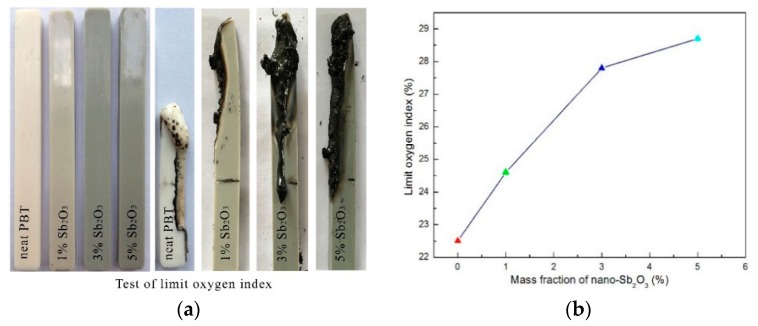
LOI of the neat PBT and its composites. (**a**) images of burnt samples before and after LOI test (**b**) curve of limit oxgen index with different mass fraction of nano-Sb_2_O_3._

As simple and important methods for evaluating the flame retardancy of polymeric materials, LOI testing and vertical flame testing were conducted to understand the combustion behavior of the neat PBT and its composites, and the results are presented in Table 7 and Figure 6.

The results showed that the value of LOI was 21.8% for the neat PBT, and there was a droplet which could ignite the absorbent cotton as it burned. When adding 1 wt % nano-Sb_2_O_3_ flame retardant, the value of LOI increased to 24.6% and reached UL-94 V-1 grade. By increasing the amount of flame retardant, the value of LOI showed a gradual increasing trend. When the amounts of nano-Sb_2_O_3_ flame retardant were 3 wt % and 5 wt %, the LOI value increased to 27.8% and 28.7%, reached UL-94 V-0 grade, and showed lower times of burning. The results showed that the Br–Sb synergistic flame-retardant system had good flame retardant effect. This was consistent with the test results of the cone calorimeter.

### 3.6. Discussion of the Mechanism

On the basis of all the above experimental results and discussions, the synergistic flame-retardant mechanism of nano-Sb_2_O_3_ and BEO can be elucidated as follows.

In the gas phase, the neat PBT formed active OH and H radicals during combustion. The reaction rate increased rapidly and released a large amount of heat (Figure 4a,b). Meanwhile, mass loss became greater, while, HBr, produced by pyrolysis, reacted with nano-Sb_2_O_3_, producing considerable SbBr_3_ at high temperature. The SbBr_3_ vapor could dilute ignitable gases, cut off the supply of oxygen, and take away the heat produced during combustion. Meanwhile, the decomposition of SbBr_3_ could produce bromine radicals which acted as radical scavengers [35]. The bromine radicals trapped the OH and H radicals and changed the reaction mode. In a combusting reaction zone, the oxygen radicals reacted with antimony and produced antimony oxide radicals, which also could trap active H and OH, decreasing the reaction heat [36].

In the condensed phase, all the Sb element was released to the gas phase according to the SEM–EDX tests (Table 6); however, nano-Sb_2_O_3_ synergizing with BEO could enhance the heat absorption and decrease the material surface temperature rapidly during combustion. Nano-Sb_2_O_3_ could produce the effect of catalytic cross-linking during combustion [37], increasing the amount of carbon residues and the thermal stability. This can be seen from the experimental values and theoretical values of the 3 wt % nano-Sb_2_O_3_ thermogravimetric data (Figure 3). Nano-Sb_2_O_3_ could also enhance the formation of char by the polymer, as demonstrated in the SEM images of cone calorimeter residues (Figure 5). The surface of the char residue of the neat PBT exhibited a continuous porous morphology, which indicated that a non-compact char residue was formed. The decomposition gases easily passed through the char layer into the flame zone to take part in the burning. When adding nano-Sb_2_O_3_ particles, the porous morphology of the composites obviously decreased. A more continuous and compact char residue was formed, which could efficiently prevent the decomposition gases from passing through the char layer. The compact char layers that formed could provide a thermally insulating barrier on the surface of the matrix and reduce heat and oxygen transmission into the material.

## 4. Conclusions

The incorporation of nano-Sb_2_O_3_ could improve the crystallization temperature, crystallinity, and thermal stability of the PBT matrix. SEM observations showed that the nano-Sb_2_O_3_ particles were more homogeneously distributed within the PBT matrix. The tensile strength of the PBT matrix was improved, as a result of the good dispersion and interfacial interactions between the nano-Sb_2_O_3_particles and the PBT matrix. The HRR, PHRR, THR, PCO_2_P, and PCOP were significantly reduced, with a maximum reduction of 57.6%, 75.5%, 43.7%, 76.7%, and 10.1% in the cone calorimeter tests. There was more char residue left, and the composites residue was firmer, thicker, and more compact. The limiting oxygen index and the flame-retardant grade were significantly improved. These results indicate that nano-Sb_2_O_3_ exhibited good synergistic flame-retardant properties and could effectively inhibit the rapid combustion of the PBT matrix.

## Figures and Tables

**Table 1:** Composition of the formulations (wt %). BEO, brominated epoxy resin.

Sample No.	PBT	BEO	Nano-Sb_2_O_3_
Neat PBT	100	0	0
PBT/BEO/nano-Sb_2_O_3_ 1%	83	16	1
PBT/BEO/nano-Sb_2_O_3_ 3%	81	16	3
PBT/BEO/nano-Sb_2_O_3_ 5%	79	16	5

**Table 2:** Thermodynamic parameters for the nonisothermal crystallization of neat PBT and its nanocomposites.

Sample No.	T_onset_ (°C)	T_c_ (°C)	T_onset_-T_c_/°C	∆H_m_ (J·g^−1^)	NE (%)	X_c_ (%)
Neat PBT	204.3	195.2	9.1	31.6	0	22.6
PBT/BEO/nano-Sb_2_O_3_ 1%	205.9	199.7	6.2	31.9	49.5	27.5
PBT/BEO/nano-Sb_2_O_3_ 3%	211.1	202.6	8.5	34.7	81.3	30.6
PBT/BEO/nano-Sb_2_O_3_ 5%	213.1	203.4	9.7	29.6	90.1	26.8

**Table 3:** Mechanical properties of the neat PBT and of its nanocomposites.

Sample No.	Tensile Strength (MPa)	Young’s Modulus (GPa)
Neat PBT	54.6 ± 0.5	1.8 ± 0.1
PBT/BEO/nano-Sb_2_O_3_ 1%	61.1 ± 0.8	2.1 ± 0.1
PBT/BEO/nano-Sb_2_O_3_ 3%	61.7 ± 0.7	2.3 ± 0.1
PBT/BEO/nano-Sb_2_O_3_ 5%	58.5 ± 0.7	2.2 ± 0.1

**Table 4:** Thermogravimetric analysis (TGA) data of the neat PBT and its nanocomposites under nitrogen (10 °C/min, 5–10 mg; error ± 0.5 wt %, ±1 °C).

Sample No.	T_10%_ (°C)	T_Peak%_ (°C)	Char at 600 °C (%)
Neat PBT	372	407	1.1
PBT/BEO/nano-Sb_2_O_3_ 1%	355	396	2.9
PBT/BEO/nano-Sb_2_O_3_ 3% (experimental)	357	391	11.8
PBT/BEO/nano-Sb_2_O_3_ 3% (calculated)	353	401	8.0
PBT/BEO/nano-Sb_2_O_3_ 5%	356	397	10.1

**Table 5:** Cone calorimeter data for the neat PBT and its composites at a heat flux of 50 kW/m^2^.

Sample No.	TTI (s)	HRR (kW/m^2^)	PHRR (kW/m^2^)	THR (MJ/m^2^)	PSPR (m^2^/s)	TSP (m^2^/kg)	PCO_2_P (kg/kg)	PCOP (kg/kg)	Residue (wt %)
Error	±2	±10	±10	±1	±0.01	±30	±0.01	±0.002	±0.5
Neat PBT	36	375.1	917.5 (451 s) ^a^	265.6	0.073	342	18.91	1.058	3.6
PBT/BEO/nano-Sb_2_O_3_ 1%	25	203.2	289.0 (789 s) ^a^	189.4	0.210	662	6.48	0.683	7.7
PBT/BEO/nano-Sb_2_O_3_ 3%	22	161.1	224.5 (841 s) ^a^	154.2	0.241	691	6.06	0.965	11.3
PBT/BEO/nano-Sb_2_O_3_ 5%	20	159.4	230.6 (616 s) ^a^	149.5	0.330	698	4.41	0.951	10.5

TTI: time to ignition; HRR: average heat release rate; PHRR: peak heat release rate; THR: total heat release; PSPR: average peak smoke production rate; TSP: total smoke production; PCO_2_P: peak CO_2_ production; PCOP: peak CO production; ^a^: time to peak heat release rate.

**Table 6:** Energy dispersive x-ray (EDX) data of the residues for the neat PBT and its composites after the cone calorimeter tests.

Sample No.	Element Content (wt %)		
	C	O	Sb
Neat PBT	95.69	4.31	0
PBT/BEO/Sb_2_O_3_ 1%	95.91	4.09	0
PBT/BEO/Sb_2_O_3_ 3%	100	0	0
PBT/BEO/Sb_2_O_3_ 5%	100	0	0

**Table 7:** Results of the limiting oxygen index (LOI) and UL-94 tests for the neat PBT and its composites.

Sample	LOI (%)	UL94, 4.0 mm Bar		
		t_1_/t_2_^a^ (s)	Dripping	Rating
Neat PBT	21.8 ± 1	BC ^b^	Yes	NR ^c^
PBT/BEO/nano-Sb_2_O_3_ 1%	24.6 ± 1	11.7/13.8	No	V-1
PBT/BEO/nano-Sb_2_O_3_3%	27.8 ± 1	5.1/7.6	No	V-0
PBT/BEO/nano-Sb_2_O_3_ 5%	28.7 ± 1	3.8/5.6	No	V-0

^a^ t_1_ and t_2_, average combustion times after the first and second application of the flame. ^b^ BC, burns to clamp. ^c^ NR, not rated.

## References

[1] Yang L., Chen H., Jia S., Lu X., Huang J. (2014). Influences of ethylene-butylacrylate-glycidyl methacrylate on morphology and mechanical properties of poly(butylene erephthalate)/polyolefin elastomer blends. J. Appl. Polym. Sci..

[2] Zhang D.H., He M., He W.D., Zhou Y.Q., Shu H., Yu J. (2017). Influence of thermo-oxidative ageing on the thermal and dynamical mechanical properties of long glass fibre-reinforced poly(butylene terephthalate) composites filled with DOPO. Materials.

[3] Yuan B.L., Hu Y., Chen X.F., Shi Y.Q., Niu Y., Zhang Y., He S., Dai H.M. (2017). Dual modification of graphene by polymeric flame retardant and Ni(OH)_2_ nanosheets for improving flame retardancy of polypropylene. Compos. Part A.

[4] Qiu S.L., Wang X., Yu B., Feng X.M., Mu X.W., Richard K.K., Hu Y. (2017). Flame-retardant-wrapped polyphosphazene nanotubes: A novelstrategy for enhancing the flame retardancy and smoke toxicity suppression of epoxy resins. J. Hazard. Mater..

[5] Niroumand J.S., Peighambardoust S.J., Shenavar A. (2016). Polystyrene-based composites and nanocomposites with reduced brominated-flame retardant. Iran. Polym. J..

[6] Samyn F., Bourbigo S., Jama C., Bellayer S., Nazare S., Hull R., Fina A., Castrovinci A., Camino G. (2008). haracterisation of the dispersion in polymer flame retarded nanocomposites. Eur. Polym. J..

[7] Bakirtzis D., Ramani A., Delichatsios M.A., Zhang J. (2009). Structure of the condensed phase and char of fire-retarded PBT nanocomposites by TGA/ATR in N_2_. Fire Saf. J..

[8] Matzen M., Kandola B., Huth C., Schartel B. (2015). Influence of Flame Retardants on the Melt Dripping Behaviour of Thermoplastic Polymers. Materials.

[9] Grause G., Ishibashi J., Kameda T., Bhaskar T., Yoshioka T. (2010). Kinetic studies of the decomposition of flame retardant containing high-impact polystyrene. Polym. Degrad. Stab..

[10] Yang W., Kan Y.C., Song L., Richard K. (2011). Effect of organo-modified montmorillonite on flame retardant poly(1,4-butyleneterephthalate) composites. Polym. Adv. Technol..

[11] Gallo E., Braun U., Schartel B., Russo P., Aciernoa D. (2009). Halogen-free flame retardedpoly(butylenes terephthalate) (PBT) using metal oxides/PBT nanocompositesin combination with aluminium phosphinate. Polym. Degrad. Stab..

[12] Zhu S.E., Wang L.L., Chen H., Yang W., Yue A.C., Chen T.B., Luo C., Bin W.M. (2018). Comparative Studies on Thermal, Mechanical, and Flame Retardant Properties of PBT Nanocomposites via Different Oxidation State Phosphorus-Containing Agents Modified Amino-CNTs. Nanomaterials.

[13] Xu J.L., Zhou S.G., Niu L., Wen C. (2016). Effect of Sb_2_O_3_ Modified by Various Surface Active Agents on Flame Retardant Properties of PVC Composite. J. Mater. Eng..

[14] Xu C.H., Shi S.Q., Surya C., Woo C.H. (2007). Synthesis of antimony oxide nano-particles by vapor transport and condensation. J. Mater. Sci..

[15] Xu J.L., Zhang L., Guo Q., Feng C. (2014). Research on the preparation of antimony nanoparticles by mechanical ball milling. Key Eng. Mater..

[16] Yang W.H., Xu J.L., Niu L., Kang C.H., Ma B.X. (2017). Analysis of agglomeration and interfacial properties in PBT/nano-Sb_2_O_3_ composites. J. Adhes. Sci. Technol..

[17] Si M.M., Feng J., Hao J.W., Xu L.S., Du J.X. (2014). Synergistic flame retardant effects and mechanisms of nano-Sb_2_O_3_ in combination with aluminum phosphinate in poly(ethylene terephthalate). Polym. Degrad. Stab..

[18] Huang G.B., Song P.A., Liu L.N. (2016). Fabrication of multifunctional graphene decorated with bromine and nano-Sb_2_O_3_ towards high-performance polymer nanocomposites. Carbon.

[19] Mirdamadian Z., Ghanbari D. (2014). Synergistic Effect between Sb_2_O_3_ Nanoparticles–Trichloromelamine and Carbon Nanotube on the Flame Retardancy and Thermal Stability of the Cellulose Acetate. J. Clust. Sci..

[20] Yang W.H., Xu J.L., Niu L., Kang C.H., Ma B.X. (2018). Effects of high energy ball milling on mechanicaland interfacial properties of PBT/nano-Sb_2_O_3_ composites. J. Adhes. Sci. Technol..

[21] (2009). Plastics-Determination of Burning Behaviour by Oxygen Index-Part 2: Ambient-Temperature Test.

[22] (2015). Reaction-to-Fire Test-Heat Release, Smoke Production and Mass Loss Rate.

[23] Chen S.J., Jin J., Zhang J. (2011). Non-isothermal crystallization behaviors of poly(4-methyl-pentene-1). J. Therm. Anal. Calorim..

[24] Yang W., Zhou H., Yang B.H., Lu H.D., Song L., Hu Y. (2016). Facile preparation of modified carbon nanotube-reinforced PBT nanocomposites with enhanced thermal, flame retardancy, and mechanical properties. Polym. Compos..

[25] Huang T., Li J.L., Yang J.H., Zhang N., Wang Y., Zhou Z.W. (2018). Carbon nanotubes induced microstructure and property changes ofpolycarbonate/poly(butylene terephthalate) blend. Compos. Part B.

[26] Yin H.J., Dittrich B., Farooq M., Schartel B. (2015). Carbon-based nanofillers/Poly(butylene terephthalate): Thermal, dielectric, electrical and rheological properties. J. Polym. Res..

[27] Righetti M.C., Di Lorenzo M.L. (2004). Melting process of poly (butylene terephthalate) analyzed by temperature-modulated differential scanning calorimetry. J. Polym. Sci. Part B.

[28] Wu D.F., Wu L.C., Yu G.C., Xu B., Zhang M. (2008). Crystallization and thermal behavior of multiwalled carbon nanotube/poly(butylenes terephthalate) composites. Polym. Eng. Sci..

[29] Deshmukh G.S., Peshwe D.R., Pathak S.U., Ekhe J. (2011). A study on effect of mineral additions on the mechanical, thermal, and structural properties of poly(butylene terephthalate) (PBT) composites. J. Polym. Res..

[30] Abe H. (2006). Thermal Degradation of Environmentally Degradable Poly(hydroxyalkanoic acid)s. Macromol. Biosci..

[31] Lu H.D., Wilkie C. (2011). Fire performance of flame retardant polypropylene and polystyrene composites screened with microscale combustion calorimetry. Polym. Adv. Technol..

[32] Wang D., Zhang Q.G., Zhou K.Q., Yang W., Hu Y., Gong X.L. (2014). The influence of manganese–cobalt oxide/graphene on reducing firehazards of poly(butylene terephthalate). J. Hazard. Mater..

[33] Nazare S., Kandola B.K., Horrocks A.R. (2008). Smoke, CO, and CO_2_ Measurements and Evaluationusing Different Fire Testing Techniques for Flame Retardant Unsaturated Polyester Resin Formulations. J. Fire Sci..

[34] Guo X., Wang H., Ma D.L., He J.N., Lei Z.Q. (2018). Synthesis of a novel, multifunctional inorganic curing agent and its effect on the flame-retardant and mechanical properties of intrinsically flame retardant epoxy resin. J. Appl. Polym. Sci..

[35] Zhang J.L., Li G.Y., Wu Q.L., Li M.C., Sun X.X., Ring D. (2017). Synergistic influence of halogenated flame retardants and nanoclay on flame performance of high density polyethylene and wood flour composites. RSC Adv..

[36] Babushok V., Deglmann P., Krämer R., Linteris G.T. (2017). Influence of Antimony-Halogen Additives on Flame Propagation. Combust. Sci. Technol..

[37] Gallo E., Schartel B., Acierno D., Russo P. (2011). Flame retardant biocomposites: Synergism between phosphinate and nanometric metal oxides. Eur. Polym. J..

